# 1,1′-(*p*-Phenyl­enedimethyl­ene)dipiperidin-4-one

**DOI:** 10.1107/S1600536809052908

**Published:** 2009-12-16

**Authors:** V. Vijayakumar, K. Rajesh, J. Suresh, T. Narasimhamurthy, P. L. Nilantha Lakshman

**Affiliations:** a Organic Chemistry Division, School of Advanced Sciences, VIT University, Vellore 632 014, India; b Department of Physics, The Madura College, Madurai 625 011, India; c Materials Research Centre, Indian Institute of Science, Bangalore 560 012, India; d Department of Food Science and Technology, Faculty of Agriculture, University of Ruhuna, Mapalana, Kamburupitiya 81100, Sri Lanka

## Abstract

In the mol­ecule of the title compound, C_18_H_24_N_2_O_2_, the piperidine rings are in chair conformations. The crystal structure is stabilized by inter­molecular C—H⋯O hydrogen bonding. There are neither C—H⋯π nor π–π inter­actions in the structure.

## Related literature

For hydrogen-bond motifs, see: Bernstein *et al.* (1995 ▶). For ring puckering parameters, see Cremer & Pople (1975 ▶).
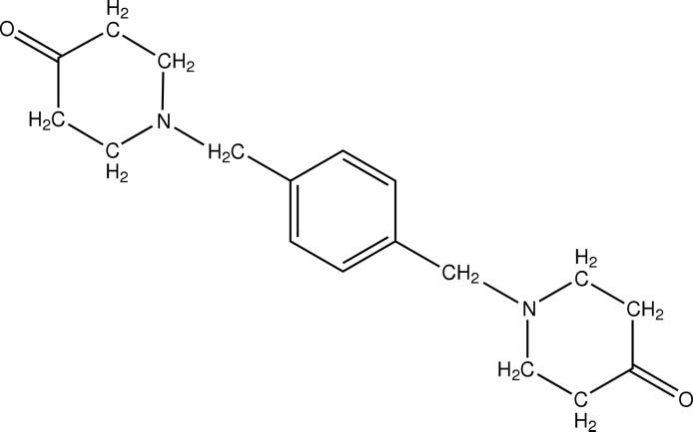


## Experimental

### 

#### Crystal data

C_18_H_24_N_2_O_2_
*M* *_r_* = 300.39
Monoclinic, 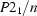

*a* = 6.2701 (5) Å
*b* = 8.0990 (6) Å
*c* = 15.8978 (13) Å
β = 98.275 (2)°
*V* = 798.91 (11) Å^3^
*Z* = 2
Mo *K*α radiation
μ = 0.08 mm^−1^
*T* = 293 K
0.19 × 0.17 × 0.15 mm

#### Data collection

Bruker SMART APEX CCD diffractometer
Absorption correction: multi-scan (*SADABS*; Bruker, 1998 ▶) *T*_min_ = 0.984, *T*_max_ = 0.987
4782 measured reflections
1826 independent reflections
1424 reflections with *I* > 2σ(*I*)
*R*_int_ = 0.014

#### Refinement

*R*[*F* ^2^ > 2σ(*F* ^2^)] = 0.043
*wR*(*F* ^2^) = 0.123
*S* = 1.05
1826 reflections
100 parameters
H-atom parameters constrained
Δρ_max_ = 0.20 e Å^−3^
Δρ_min_ = −0.14 e Å^−3^

### 

Data collection: *SMART* (Bruker, 2001 ▶); cell refinement: *SAINT* (Bruker, 2001 ▶); data reduction: *SAINT*; program(s) used to solve structure: *SHELXS97* (Sheldrick, 2008 ▶); program(s) used to refine structure: *SHELXL97* (Sheldrick, 2008 ▶); molecular graphics: *PLATON* (Spek, 2009 ▶); software used to prepare material for publication: *SHELXL97*.

## Supplementary Material

Crystal structure: contains datablocks global, I. DOI: 10.1107/S1600536809052908/bt5127sup1.cif

Structure factors: contains datablocks I. DOI: 10.1107/S1600536809052908/bt5127Isup2.hkl

Additional supplementary materials:  crystallographic information; 3D view; checkCIF report

## Figures and Tables

**Table 1:** Hydrogen-bond geometry (Å, °)

*D*—H⋯*A*	*D*—H	H⋯*A*	*D*⋯*A*	*D*—H⋯*A*
C7—H7*B*⋯O1^i^	0.97	2.56	3.2235 (17)	126

Symmetry code: (i) 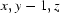.

**Fig. 1.**
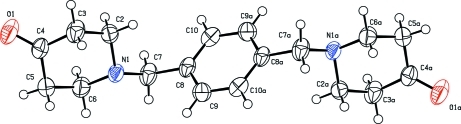
The molecular structure of title compound with atom numbering scheme and 50% probability displacement ellipsoids.

## supplementary crystallographic information

### Comment

The configuration and conformation of the title compound, (I) and the atom numbering scheme are shown in the *ORTEP* drawing (Fig. 1). The piperidone ring exibits chair conformation as evident from the puckering parameters (Q)=0.549 (1) Å, θ = 173.4 (2) °, ψ = 181.9 (1) ° (Cremer & Pople, 1975).

In the crystal structure, an intermolecular C—H···O bond is found generating R22(24) motif (Bernstein *et al.*,  1995).

### Experimental

A mixture of 4-piperidone monohydrate hydrochloride (2 mol), 1,4-bis(bromomethyl)benzene (1 mol) and potassium carbonate (6 mol) in anhydrous benzene was refluxed for 7 h. The completion of reaction was monitored by TLC. Potassium carbonate was filtered off and the excess solvent was removed under reduced pressure. The solid obtained was purified over a column of silica gel (60–120 mesh size) using benzene-ethyl acetate (60–80 °C) in the ratio of 20:80. Yield: 40% m.p. 289°C.

### Refinement

The H atoms were placed in calculated positions and allowed to ride on their carrier atoms with C—H = 0.93–0.97 Å.*U*_iso_ = 1.2*U*_eq_(C) for CH and CH_2_ groups.

### Crystal data

**Table:**

C_18_H_24_N_2_O_2_	*F*(000) = 324
*M**_r_* = 300.39	*D*_x_ = 1.249 Mg m^−^^3^
Monoclinic, *P*2_1_/*n*	Mo *K*α radiation, λ = 0.71073 Å
Hall symbol: -P 2yn	Cell parameters from 2500 reflections
*a* = 6.2701 (5) Å	θ = 2–30°
*b* = 8.0990 (6) Å	µ = 0.08 mm^−^^1^
*c* = 15.8978 (13) Å	*T* = 293 K
β = 98.275 (2)°	Block, colourless
*V* = 798.91 (11) Å^3^	0.19 × 0.17 × 0.15 mm
*Z* = 2	

### Data collection

**Table:**

Bruker SMART APEX CCD diffractometer	1826 independent reflections
Radiation source: fine-focus sealed tube	1424 reflections with *I* > 2σ(*I*)
graphite	*R*_int_ = 0.014
ω scans	θ_max_ = 27.5°, θ_min_ = 2.6°
Absorption correction: multi-scan (*SADABS*; Bruker, 1998)	*h* = −8→4
*T*_min_ = 0.984, *T*_max_ = 0.987	*k* = −10→10
4782 measured reflections	*l* = −20→19

### Refinement

**Table:**

Refinement on *F*^2^	Primary atom site location: structure-invariant direct methods
Least-squares matrix: full	Secondary atom site location: difference Fourier map
*R*[*F*^2^ > 2σ(*F*^2^)] = 0.043	Hydrogen site location: inferred from neighbouring sites
*wR*(*F*^2^) = 0.123	H-atom parameters constrained
*S* = 1.05	*w* = 1/[σ^2^(*F*_o_^2^) + (0.0639*P*)^2^ + 0.1074*P*] where *P* = (*F*_o_^2^ + 2*F*_c_^2^)/3
1826 reflections	(Δ/σ)_max_ < 0.001
100 parameters	Δρ_max_ = 0.20 e Å^−^^3^
0 restraints	Δρ_min_ = −0.14 e Å^−^^3^

### Special details

**Table:**

Geometry. All e.s.d.'s (except the e.s.d. in the dihedral angle between two l.s. planes) are estimated using the full covariance matrix. The cell e.s.d.'s are taken into account individually in the estimation of e.s.d.'s in distances, angles and torsion angles; correlations between e.s.d.'s in cell parameters are only used when they are defined by crystal symmetry. An approximate (isotropic) treatment of cell e.s.d.'s is used for estimating e.s.d.'s involving l.s. planes.
Refinement. Refinement of *F*^2^ against ALL reflections. The weighted *R*-factor *wR* and goodness of fit *S* are based on *F*^2^, conventional *R*-factors *R* are based on *F*, with *F* set to zero for negative *F*^2^. The threshold expression of *F*^2^ > σ(*F*^2^) is used only for calculating *R*-factors(gt) *etc*. and is not relevant to the choice of reflections for refinement. *R*-factors based on *F*^2^ are statistically about twice as large as those based on *F*, and *R*- factors based on ALL data will be even larger.

### Fractional atomic coordinates and isotropic or equivalent isotropic displacement parameters (Å^2^)

**Table:**

	*x*	*y*	*z*	*U*_iso_*/*U*_eq_	
C2	0.2931 (2)	0.43945 (15)	0.38729 (9)	0.0451 (3)	
H2A	0.3278	0.4330	0.3299	0.054*	
H2B	0.4196	0.4060	0.4260	0.054*	
C3	0.2358 (2)	0.61783 (16)	0.40660 (10)	0.0544 (4)	
H3A	0.2191	0.6276	0.4661	0.065*	
H3B	0.3519	0.6905	0.3959	0.065*	
C4	0.0312 (2)	0.66908 (16)	0.35253 (8)	0.0440 (3)	
C5	−0.1488 (2)	0.54905 (18)	0.35354 (11)	0.0584 (4)	
H5A	−0.2680	0.5796	0.3105	0.070*	
H5B	−0.1991	0.5528	0.4084	0.070*	
C6	−0.0757 (2)	0.37490 (18)	0.33662 (10)	0.0544 (4)	
H6A	−0.1918	0.2982	0.3419	0.065*	
H6B	−0.0434	0.3682	0.2789	0.065*	
C7	0.1742 (2)	0.15730 (16)	0.37561 (9)	0.0493 (4)	
H7A	0.2250	0.1575	0.3208	0.059*	
H7B	0.0458	0.0891	0.3705	0.059*	
C8	0.3452 (2)	0.07991 (14)	0.44028 (8)	0.0405 (3)	
C9	0.3083 (2)	0.05404 (16)	0.52331 (8)	0.0450 (3)	
H9	0.1799	0.0900	0.5400	0.054*	
C10	0.5395 (2)	0.02482 (16)	0.41824 (8)	0.0444 (3)	
H10	0.5682	0.0411	0.3631	0.053*	
N1	0.11571 (16)	0.32682 (12)	0.39582 (7)	0.0395 (3)	
O1	0.01374 (19)	0.79483 (13)	0.31091 (7)	0.0657 (4)	

### Atomic displacement parameters (Å^2^)

**Table:**

	*U*^11^	*U*^22^	*U*^33^	*U*^12^	*U*^13^	*U*^23^
C2	0.0372 (6)	0.0349 (7)	0.0605 (8)	−0.0033 (5)	−0.0021 (5)	0.0041 (6)
C3	0.0595 (8)	0.0329 (7)	0.0659 (9)	−0.0062 (6)	−0.0079 (7)	0.0018 (6)
C4	0.0549 (8)	0.0322 (6)	0.0448 (7)	0.0048 (5)	0.0072 (6)	0.0015 (5)
C5	0.0415 (7)	0.0509 (9)	0.0821 (10)	0.0057 (6)	0.0064 (7)	0.0227 (7)
C6	0.0423 (7)	0.0422 (8)	0.0731 (10)	−0.0085 (6)	−0.0107 (7)	0.0130 (7)
C7	0.0586 (8)	0.0308 (7)	0.0540 (8)	−0.0024 (6)	−0.0075 (6)	−0.0006 (6)
C8	0.0502 (7)	0.0233 (5)	0.0464 (7)	−0.0019 (5)	0.0015 (5)	−0.0002 (5)
C9	0.0462 (7)	0.0374 (7)	0.0528 (8)	0.0024 (5)	0.0113 (6)	−0.0021 (5)
C10	0.0573 (8)	0.0368 (7)	0.0401 (6)	−0.0039 (6)	0.0106 (6)	0.0016 (5)
N1	0.0389 (5)	0.0288 (5)	0.0484 (6)	−0.0026 (4)	−0.0022 (4)	0.0059 (4)
O1	0.0827 (8)	0.0385 (6)	0.0723 (7)	−0.0004 (5)	−0.0012 (6)	0.0175 (5)

### Geometric parameters (Å, °)

**Table:**

C2—N1	1.4598 (16)	C6—H6A	0.9700
C2—C3	1.5304 (18)	C6—H6B	0.9700
C2—H2A	0.9700	C7—N1	1.4685 (17)
C2—H2B	0.9700	C7—C8	1.5104 (18)
C3—C4	1.4964 (19)	C7—H7A	0.9700
C3—H3A	0.9700	C7—H7B	0.9700
C3—H3B	0.9700	C8—C9	1.3884 (18)
C4—O1	1.2109 (16)	C8—C10	1.3888 (19)
C4—C5	1.492 (2)	C9—C10^i^	1.3882 (18)
C5—C6	1.519 (2)	C9—H9	0.9300
C5—H5A	0.9700	C10—C9^i^	1.3882 (18)
C5—H5B	0.9700	C10—H10	0.9300
C6—N1	1.4670 (16)		
			
N1—C2—C3	111.55 (11)	C5—C6—H6A	109.2
N1—C2—H2A	109.3	N1—C6—H6B	109.2
C3—C2—H2A	109.3	C5—C6—H6B	109.2
N1—C2—H2B	109.3	H6A—C6—H6B	107.9
C3—C2—H2B	109.3	N1—C7—C8	114.47 (10)
H2A—C2—H2B	108.0	N1—C7—H7A	108.6
C4—C3—C2	110.67 (11)	C8—C7—H7A	108.6
C4—C3—H3A	109.5	N1—C7—H7B	108.6
C2—C3—H3A	109.5	C8—C7—H7B	108.6
C4—C3—H3B	109.5	H7A—C7—H7B	107.6
C2—C3—H3B	109.5	C9—C8—C10	117.61 (11)
H3A—C3—H3B	108.1	C9—C8—C7	120.72 (12)
O1—C4—C5	123.01 (13)	C10—C8—C7	121.59 (12)
O1—C4—C3	123.37 (13)	C10^i^—C9—C8	120.89 (12)
C5—C4—C3	113.62 (11)	C10^i^—C9—H9	119.6
C4—C5—C6	110.77 (12)	C8—C9—H9	119.6
C4—C5—H5A	109.5	C9^i^—C10—C8	121.50 (12)
C6—C5—H5A	109.5	C9^i^—C10—H10	119.3
C4—C5—H5B	109.5	C8—C10—H10	119.3
C6—C5—H5B	109.5	C2—N1—C6	109.77 (10)
H5A—C5—H5B	108.1	C2—N1—C7	110.25 (11)
N1—C6—C5	111.90 (12)	C6—N1—C7	108.36 (10)
N1—C6—H6A	109.2		
			
N1—C2—C3—C4	−54.57 (16)	C7—C8—C9—C10^i^	176.62 (11)
C2—C3—C4—O1	−129.37 (15)	C9—C8—C10—C9^i^	0.3 (2)
C2—C3—C4—C5	49.69 (17)	C7—C8—C10—C9^i^	−176.59 (12)
O1—C4—C5—C6	129.33 (15)	C3—C2—N1—C6	59.82 (15)
C3—C4—C5—C6	−49.73 (18)	C3—C2—N1—C7	179.12 (11)
C4—C5—C6—N1	54.57 (17)	C5—C6—N1—C2	−60.02 (16)
N1—C7—C8—C9	62.64 (17)	C5—C6—N1—C7	179.53 (12)
N1—C7—C8—C10	−120.58 (14)	C8—C7—N1—C2	69.70 (15)
C10—C8—C9—C10^i^	−0.3 (2)	C8—C7—N1—C6	−170.15 (12)

Symmetry codes: (i) −*x*+1, −*y*, −*z*+1.

### Hydrogen-bond geometry (Å, °)

**Table:**

*D*—H···*A*	*D*—H	H···*A*	*D*···*A*	*D*—H···*A*
C7—H7B···O1^ii^	0.97	2.56	3.2235 (17)	126

Symmetry codes: (ii) *x*, *y*−1, *z*.
